# From histopathology to molecular taxonomy: predicting pituitary neuroendocrine tumors invasiveness

**DOI:** 10.3389/fendo.2026.1907825

**Published:** 2026-10-27

**Authors:** Boyuan Zhang, Yuqi Sun, Hongyu Guo, XiLe Pei, Chunfa Wu, Yuanda Yang, Yuzhen Yang, Dahai Wan

**Affiliations:** 1 The First Clinical Medical College of Shanxi Medical University, Taiyuan, China; 2 Department of Neurosurgery, First Hospital of Shanxi Medical University, Taiyuan, Shanxi, China

**Keywords:** DNA methylation, liquid biopsy, molecular biomarkers, non-coding RNA, pituitary neuroendocrine tumors

## Abstract

Pituitary neuroendocrine tumors (PitNETs) are among the most prevalent intracranial tumors in adults. Although most follow an indolent clinical course, a substantial subset exhibits local invasion, which can limit gross total resection and increase the risk of residual disease and recurrence. Importantly, anatomical invasiveness should not be equated with clinical aggressiveness. Aggressive PitNETs may also exhibit a longitudinal pattern of repeated or multiple local recurrences and frequently require multiple therapeutic interventions. The 2022 Fifth Edition of the WHO Classification formally renamed pituitary adenomas to PitNETs and established a lineage classification framework centered on core transcription factors, including PIT1, TPIT, and SF1, thereby driving the transition from traditional pathological diagnosis to molecular subtyping. Driven by recent advances in high-throughput sequencing, liquid biopsy, and single-cell omics, numerous novel molecular biomarkers have emerged, enabling the early pre- and postoperative identification of invasive subpopulations. This review systematically outlines recent progress in multidimensional biomarkers—including DNA methylation, protein markers, non-coding RNAs, liquid biopsy indicators, and tumor microenvironmental features—to provide an updated synthesis of candidate biomarkers that may support future risk stratification and individualized management of invasive PitNETs.

## 1 Introduction

Pituitary neuroendocrine tumors (PitNETs) account for approximately 10% to 15% of all intracranial tumors, representing the third most common primary central nervous system tumor (1). In 2022, the WHO classification formally renamed pituitary adenomas to PitNETs, marking a paradigm shift toward precise diagnostic classification based on transcription factor immunohistochemistry (IHC) (2, 3). This updated framework primarily categorizes PitNETs into three major lineages—PIT1, TPIT, and SF1—alongside a minority of tumors that lack distinct lineage markers (4). A critical challenge in current clinical practice is the early identification of PitNETs at increased risk of local invasion, recurrence, progression, or clinically aggressive behavior. These endpoints should be clearly distinguished. Invasiveness refers primarily to anatomical extension into surrounding structures, whereas aggressiveness represents a longitudinal clinical phenotype characterized by unusually rapid progression or clinically relevant progression despite optimal standard therapies and is frequently accompanied by multiple local recurrences. Accordingly, throughout this review, we preserve the terminology used in the original studies: cohorts defined by anatomical or radiological invasion are described as invasive, whereas tumors explicitly classified as aggressive or fulfilling criteria for aggressive pituitary tumors are described as aggressive. Emerging evidence suggests that PitNET invasiveness is driven by a complex interplay of genetic alterations, epigenetic dysregulation, tumor stemness, and microenvironmental remodeling. The principal biomarkers associated with tumor invasion, recurrence, and prognosis are summarized in **Table 1**. To place these advances in context, the following section briefly traces the evolution of PitNET assessment from morphology and anatomical invasion grading to clinicopathological risk stratification, lineage-based classification, molecular taxonomy, and emerging multimodal prediction, together with the parallel refinement of the concept of aggressive pituitary tumors.

**Table 1:** Summary of candidate molecular, cellular, and microenvironmental biomarkers associated with invasiveness, recurrence, and prognosis in PitNETs.

Core molecules/features	Sample type	Predictive/clinical value	AUC/effect size	Ref.
*USP8* (hotspot region in 14-3–3 binding motif)	Tumor tissue	Favorable prognosis in corticotroph PitNETs	OR = 0.38 (for invasiveness)	(28, 29)
*TP53* mutation	Tumor tissue	Invasiveness and shortened overall survival	~24% in invasive corticotroph PitNETs	(21, 28)
*ATRX* (mutation and loss of nuclear immunohistochemical staining)	Tumor tissue	Associated with invasive or metastatic phenotypes; candidate risk-enrichment marker	—	(22, 23)
*SF3B1* R625H	Tumor tissue	Invasiveness and shortened DFS/OS in lactotroph PitNETs	≤42% in metastatic lactotroph PitNETs	(24, 25)
*MIR4535/SLC8A1-AS1/TTC34* panel	Plasma cfDNA	Differentiation of invasive tumors	AUC = 0.980	(32)
Genome-wide methylation profiling + copy number variation (CNV)	Tumor tissue	Stratification of benign, invasive, and metastatic phenotypes	Distinct three-cluster separation	(30)
*CHST7* promoter methylation/*CHST7* expression	Tumor tissue	Associated with lineage differentiation and proliferative activity; not validated as a predictor of clinically defined aggressiveness	—	(31)
*GALR1* ↓, *PENK* ↓	Tumor tissue	Tumor-suppressive markers indicating invasiveness upon downregulation	—	(35)
*miR-*34a	Serum and tissue	Octreotide resistance	—	(44)
*miR-*137	Serum and tissue	Suppression of lactotroph PitNET progression	—	(45)
*miR-*423-5p	Serum	Diagnosis of somatotroph PitNETs	—	(5)
*MEG3*	Tumor tissue	Suppression of invasiveness (specifically in *GNAS*-mutant types)	—	(55)
*Lnc H19*	Tumor tissue/Exosomes	lactotroph PitNETs suppression and sensitization to dopamine agonists	—	(7)
*circMFN2* (*miR-*146a-3p/TRAF6/NF-κB axis)	Serum exosomes	Tumor invasiveness	—	(58)
*miR-*486-5p	Serum exosomes	Prediction of recurrence in non-functioning PitNETs	—	(5)
Cyclin D1 ≥ 10% + P53 < 10%	Tumor tissue	Prediction of tumor invasiveness	Reported 100% specificity/accuracy in the original study cohort	(16)
PD-L1 ≥ 1%	Tumor tissue	Lineage-associated candidate immune biomarker	OR = 2.35 reported in a single cohort, not independently validated	(16)
Collagen-1	Tumor tissue	Prediction of recurrence	AUC = 0.88	(8)
MMR pathway (*MSH2, MSH6, MLH1, PMS2*)	Tumor tissue/transcriptomic data	Lineage-dependent association with invasiveness	—	(36)
CD8^+^T-cell infiltration	Tumor tissue/single-cell transcriptomic data	Associated with reduced invasiveness	—	(37)
TrkAIII alternative splicing/TrkAIII:fs-TrkA ratio	Tumor tissue	Associated with invasiveness, particularly in PIT1-lineage tumors	—	(43)

AUC, area under the receiver operating characteristic curve; cfDNA, cell-free DNA; CNV, copy number variation; DFS, disease-free survival; EMT, epithelial-mesenchymal transition; GNAS, Gs alpha subunit gene; lncRNA, long non-coding RNA; ncRNA, non-coding RNA; MMR, mismatch repair; OR, odds ratio; OS, overall survival; PitNET, pituitary neuroendocrine tumor; Ref, references.

↓ indicates downregulation or decreased expression.

## 2 Current landscape and unmet needs in molecular prediction of PitNET invasiveness

Recent studies have substantially advanced the understanding of individual components of the molecular landscape of PitNETs, including liquid biopsy (5), non-coding and exosomal RNAs (6, 7), extracellular-matrix and collagen remodeling (8), and biomarker discovery in non-functioning PitNETs (9). Nevertheless, several important gaps remain in translating these rapidly expanding molecular data into clinically meaningful risk stratification.

Much of the existing literature remains organized according to individual biomarker classes, biological processes, or specific tumor subtypes rather than according to clinically relevant outcomes. In particular, molecular classification, local invasiveness, recurrence or progression, treatment resistance, and clinically defined aggressiveness are frequently discussed within the same conceptual framework despite representing distinct biological and clinical endpoints. This distinction has become increasingly important as recent genomic, epigenomic, transcriptomic, circulating-biomarker, and tumor-microenvironment studies have revealed substantial heterogeneity both within and across conventional PitNET lineages.

The recent systematic review by Tajuddin et al. comprehensively summarized biomarker studies in non-functioning PitNETs, including invasion- and epithelial–mesenchymal transition-related markers, non-coding RNA and liquid-biopsy candidates, and multi-omic and epigenetic signatures (9). However, the available evidence extends beyond non-functioning tumors and increasingly includes lineage-specific genomic alterations, molecular taxonomy, treatment-response biomarkers, circulating metabolites, and immune-microenvironment features across functioning and non-functioning PitNETs. Accordingly, there remains a need to integrate these heterogeneous molecular findings within a framework that considers tumor lineage, biological phenotype, clinical endpoint, and level of validation.

A conceptual distinction should therefore be made between molecular taxonomy and molecular prognostication throughout this review. Molecular taxonomy refers to the classification of PitNETs according to lineage, cellular identity, and integrated molecular characteristics, whereas molecular prognostication concerns the ability of specific biomarkers or molecular signatures to predict clinically relevant outcomes, including local invasion, recurrence or progression, treatment resistance, and clinically aggressive behavior. Although these dimensions may overlap, they are not interchangeable. Accordingly, the remainder of this review is organized along a biological continuum, progressing from lineage-based and molecular taxonomy to tumor-intrinsic genomic, epigenetic, and transcriptomic alterations; downstream signaling and invasive cellular programs; microenvironmental remodeling; and, finally, circulating biomarkers and multimodal approaches for clinical risk stratification. This structure is intended to distinguish molecular classification from mechanistic drivers and clinically oriented prognostic biomarkers while minimizing overlap among biomarker categories.

## 3 Histopathological classification framework of PitNETs in the 2022 WHO classification and its molecular extensions

### 3.1 Lineage-defining transcription factors in histopathological classification

The 2022 WHO classification of PitNETs is primarily organized according to adenohypophyseal cell lineage and integrates tumor morphology, pituitary hormone expression, and immunohistochemical assessment of lineage-defining transcription factors. PIT1, TPIT, and SF1 therefore serve as key histopathological markers for assigning most PitNETs to PIT1-, TPIT-, and SF1-lineage categories, respectively. This lineage-based approach represents an important refinement of conventional classification based predominantly on hormonal phenotype and morphology but should not be regarded as molecular typing in the strict genomic sense (2, 4). Beyond their diagnostic role, lineage assignment may also provide clinically relevant information. In a clinical cohort of non-functioning PitNETs, SF1-lineage tumors constituted the largest subgroup (58.4%) and were associated with a relatively favorable outcome, whereas TPIT-lineage tumors accounted for 18.6%. Tumors without clearly identifiable lineage characteristics (16.8%) were independently associated with postoperative residual disease or recurrence (HR = 3.02). These observations suggest that lineage-based histopathological classification may contribute to prognostic stratification, although lineage assignment itself should be distinguished from molecular predictors of invasive biological behavior (10).

### 3.2 Pangenomic classification extends the lineage-based framework

Beyond the routine histopathological lineage classification adopted by the 2022 WHO framework, integrated multi-omic analyses have provided complementary molecular stratification of PitNETs. Rather than replacing histopathological diagnosis, these approaches refine the biological taxonomy of PitNETs by identifying genomic, epigenomic, transcriptomic, and miRNA-defined heterogeneity within and across conventional lineages. In a landmark pangenomic study of 134 PitNETs, Neou et al. (11)integrated somatic mutations, chromosomal alterations, DNA methylation, transcriptomic profiles, and miRNA expression and demonstrated that pituitary cell lineage was the principal determinant of unsupervised molecular classification, preceding clinical aggressiveness as a source of molecular variation. Notably, PIT1-lineage tumors were characterized by diffuse DNA hypomethylation, increased transposable-element expression, and chromosomal instability, although this pattern was less pronounced in *GNAS*-mutated somatotroph tumors.

The study further demonstrated substantial molecular heterogeneity within conventionally defined lineages. Corticotroph tumors could be separated into molecularly distinct groups, including *USP8*-mutated overtly secreting tumors, *USP8*-wild-type tumors showing increased invasive behavior and enrichment of epithelial–mesenchymal transition signatures, and large silent corticotroph tumors displaying partial gonadotroph differentiation. Moreover, *SF1/NR5A1* expression was identified in a subset of *GNAS*-wild-type somatotroph tumors, further illustrating that lineage boundaries defined by conventional histopathology are not invariably absolute.

Independent studies have subsequently provided further support for and refinement of this lineage-centered molecular framework. Mosella et al. analyzed genome-wide DNA methylation profiles from 177 sporadic PitNETs and validated the identified methylation signatures in an independent cohort of 86 tumors, demonstrating three methylation clusters associated with adenohypophyseal cell lineage and functional status (12). Patrício da Silva-Júnior et al. further integrated methylome and transcriptome data from 77 pituitary tumors and identified molecular clusters broadly corresponding to *POU1F1*/PIT1-, *TBX19*/TPIT-, and *NR5A1*/SF1-driven lineages, while also documenting occasional discordance between clinical phenotype and lineage-marker expression, thereby emphasizing residual heterogeneity within the lineage-based framework (13). More recently, Belakhoua et al. analyzed clinical, immunohistochemical, and DNA methylation data from 118 PitNETs and demonstrated that DNA methylation profiling could distinguish the three major transcription factor-defined lineages while further resolving clinically functioning and silent subgroups within PIT1- and TPIT-lineage tumors (14). Complementing these epigenetic studies, Huang et al. used bulk and full-length single-cell RNA sequencing to characterize alternative-splicing patterns across PitNET lineages and showed that transcript-level splicing diversity provides additional resolution beyond conventional transcription factor-based classification, including the identification of a distinct TPIT-lineage subtype associated with less favorable clinical features (15). Collectively, these independent datasets support a lineage-centered molecular taxonomy while also demonstrating that substantial molecular heterogeneity persists within individual lineages and can be further resolved by different molecular platforms.

Taken together, these findings indicate that the current transcription factor-based classification is strongly supported by molecular data, while integrated molecular profiling may further resolve biologically and clinically relevant heterogeneity within and across PitNET lineages. Importantly, the principal contribution of such integrated multi-omic analyses is taxonomic: they refine tumor identity and reveal molecular heterogeneity within conventional histopathological categories. Molecular class should therefore not be regarded as synonymous with prognosis or clinically defined aggressiveness. Nevertheless, when individual molecular subgroups demonstrate reproducible associations with invasion, recurrence, progression, or treatment resistance, these taxonomic features may additionally acquire prognostic relevance. Such prognostic associations require independent clinical validation and should be distinguished from classification itself.

### 3.3 Re-evaluation of proliferative markers

Postoperative indices from surgical specimens, such as the Ki-67 proliferation index and p53 expression, reflect the proliferative activity of PitNETs and remain recommended for routine evaluation in the 2022 WHO classification (2, 4). Recent studies suggest that the combined assessment of cell-cycle-related proteins may improve risk stratification. In one reported cohort, a panel combining Cyclin D1 ≥ 10% and P53 < 10% showed high discriminatory performance for Hardy-defined invasiveness; however, further validation in independent cohorts is required before this combination can be regarded as an established predictive biomarker. Furthermore, multivariate analysis shows that a Ki-67 index ≥ 3% is significantly correlated with younger patient populations (p = 0.03), suggesting that highly proliferative tumors in young patients may possess distinct, invasive biological behaviors (16).

### 3.4 Prognostic value and clinical interpretation of the five-tier clinicopathological classification

The clinicopathological classification proposed by Trouillas et al. (17) integrates radiological invasion with proliferative parameters, including the Ki-67 index, mitotic activity, and P53 immunoreactivity, to stratify the risk of postoperative recurrence or progression. Following its initial development, the prognostic value of this system has been supported by several independent cohorts. A prospective study by Raverot et al. (18) demonstrated that Knosp Grade 2b tumors had a 3.72-fold higher risk of recurrence or progression than Knosp Grade 1a tumors, while subsequent external validation studies involving 566 and 607 surgically treated patients similarly confirmed tumor grade as an independent predictor of disease evolution and progression-free survival (17–20).Importantly, a Knosp Grade 2b classification should be regarded as a high-risk prognostic feature rather than as synonymous with clinically defined aggressiveness. Although invasive and proliferative tumors, particularly Knosp Grade 2b tumors, have an increased risk of subsequent adverse clinical evolution, not all such tumors become aggressive. According to the revised 2025 ESE guideline, an aggressive pituitary tumor should be considered when an invasive tumor demonstrates unusually rapid progression or clinically relevant progression despite optimal standard therapies. These tumors may also follow a longitudinal course characterized by multiple local recurrences and repeated therapeutic interventions. Thus, pathological grading should be interpreted together with longitudinal clinical and radiological behavior rather than being used alone to define aggressiveness.

## 4 Gene mutation biomarkers

Beyond classical proliferative markers, specific somatic alterations have emerged as candidate tissue markers that may contribute to risk stratification in PitNETs.

*TP53* mutation: Frequently detected in invasive subpopulations, its mutation rate reaches up to 24% in invasive corticotroph tumors (21). *TP53* mutations typically herald larger tumor volume, enhanced invasiveness, an elevated proliferation index, and a significantly shortened overall survival.

*ATRX* alterations: Functional inactivation of the *ATRX* gene, a key regulator of chromatin remodeling, triggers the alternative lengthening of telomeres (ALT) mechanism, thereby granting tumor cells unlimited replicative potential. This phenomenon is predominantly observed in functional corticotroph tumors and frequently co-occurs with *TP53* mutations (22, 23). Pathogenic *ATRX* alterations may result in loss of nuclear ATRX protein expression, which can be detected by immunohistochemistry. However, loss of ATRX immunoreactivity represents a protein-level surrogate of underlying *ATRX* dysfunction and should not be considered synonymous with an *ATRX* mutation. Consequently, Villa et al. recommend routine ATRX IHC screening for PitNETs with invasive features to identify high-risk patients requiring intensive follow-up (22).

*SF3B1* mutation: Notably, Li et al. were among the first to identify recurrent *SF3B1* mutations as a genomic alteration in PitNETs, providing important evidence for the involvement of aberrant RNA splicing in PitNETs (24). *SF3B1* encodes a core component of the U2 snRNP spliceosome, and its somatic hotspot mutation (R625H) is intimately linked to lactotroph PitNETs. Simon et al. reported that *SF3B1* mutations occur in approximately 3% of standard lactotroph PitNETs but soar to 42% in metastatic cases. *SF3B1*-mutated tumors display distinct aberrant splicing profiles (such as *DLG1* mis-splicing), which correlate with multiple therapeutic interventions, heightened invasiveness, and shorter disease-free and overall survival (25). Together, *TP53*, *ATRX*, and *SF3B1* alterations can be assessed in surgical tissue and may provide lineage-specific risk-enrichment information; however, their independent predictive performance for anatomical invasiveness or clinically defined aggressiveness remains unestablished.

Among these alterations, *TP53* dysfunction and ATRX loss provide a biologically coherent paired model for clonal evolution in corticotroph PitNETs. Function-altering *TP53* variants may attenuate P53 transcriptional output, weakening P21-dependent G1–S checkpoint control and PUMA/BAX-mediated mitochondrial apoptosis, thereby allowing DNA-damaged cells to survive and continue cycling. In parallel, ATRX deficiency may impair DAXX-mediated H3.3 deposition at telomeres, favoring the persistence of G-quadruplex and TERRA/R-loop structures, replication-fork stalling, and telomeric DNA breaks. In an ALT-permissive cellular context, APB-associated, break-induced-replication-like telomere synthesis may support the persistence and diversification of genomically unstable clones (21, 26, 27). Subsequent therapeutic selection may enrich resistant subclones, providing a plausible link between these molecular alterations and the evolution of aggressive clinical behavior (**Figure 1**).

**Figure 1:**
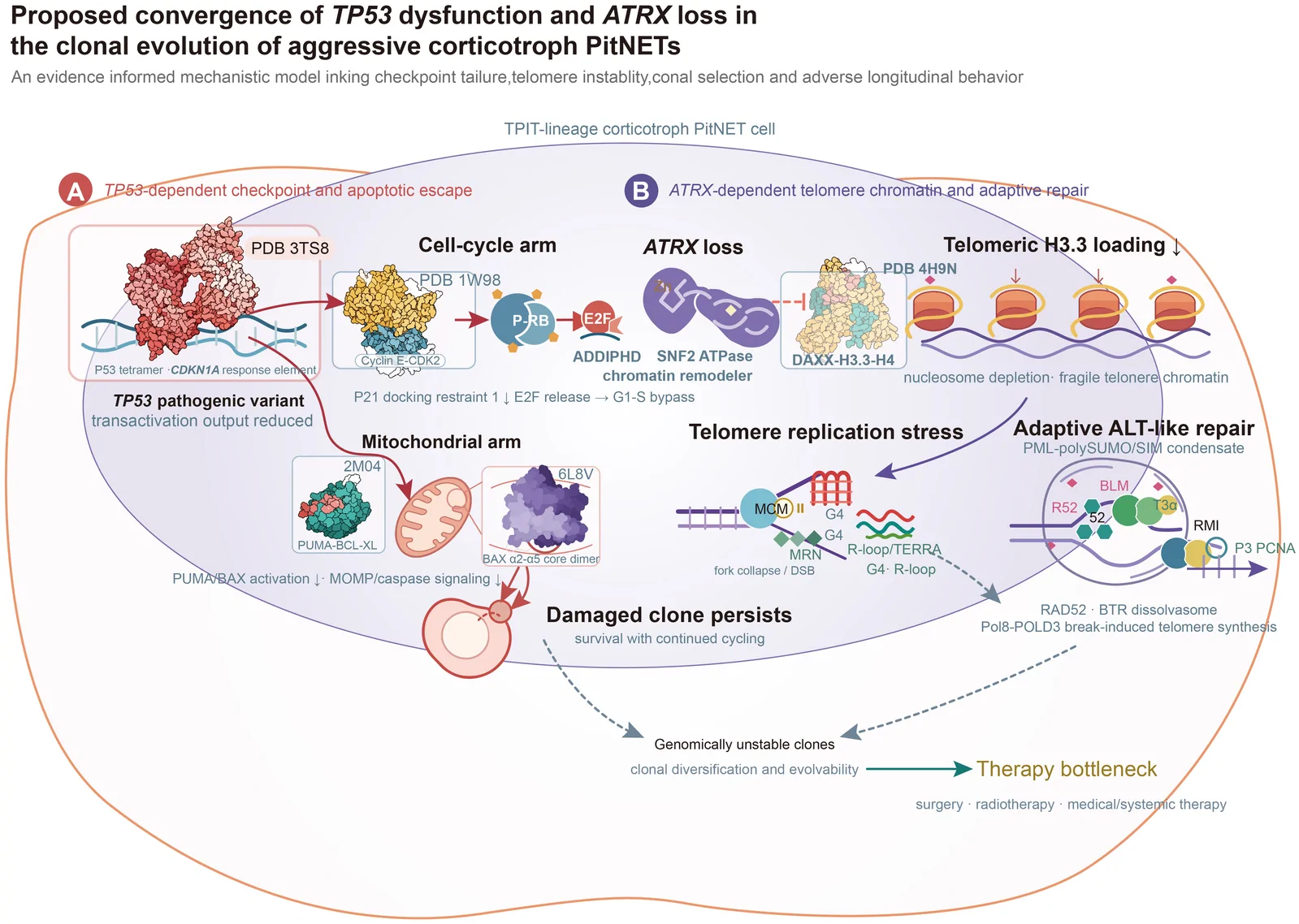
Proposed convergence of *TP53* dysfunction and ATRX loss in the clonal evolution of clinically aggressive corticotroph PitNETs. **(A)** TP53-dependent checkpoint and apoptosis escape. **(B)** ATRX-dependent telomere chromatin alterations and adaptive repair. Function-altering *TP53* variants may attenuate P53-dependent transcription, weakening P21–Cyclin E/CDK2–RB/E2F checkpoint control and PUMA–BCL-XL–BAX-mediated mitochondrial apoptosis. ATRX loss may impair DAXX-dependent H3.3 deposition at telomeres, increasing G-quadruplex- and TERRA/R-loop-associated replication stress and telomeric DNA breaks. In an ALT-permissive context, conditional APB-associated, break-induced-replication-like telomere synthesis may facilitate the persistence and diversification of genomically unstable clones. These processes may converge during treatment-mediated selection to promote persistent, recurrent, or treatment-refractory disease. Solid arrows denote well-established molecular relationships; gray dashed arrows denote PitNET-specific mechanistic inference; and the coral dashed arrow denotes clinical transition. *TP53* and *ATRX* alterations are risk-enrichment signals rather than stand-alone predictors. The figure was independently conceptualized, designed, and drawn by the authors. ALT, alternative lengthening of telomeres; APB, ALT-associated promyelocytic leukemia body; BIR, break-induced replication; DAXX, death domain-associated protein; TERRA, telomeric repeat-containing RNA.

Corticotroph adenomas possess distinct molecular genetic attributes. In a study containing 147 samples, *USP8* mutations were detected in 41% of Cushing’s disease cases but in only 8.5% of silent corticotroph tumors, while *USP48* mutations occurred in 6% of Cushing’s disease patients. Generally, *USP8*- or *USP48*-mutant tumors present with smaller volumes, exhibit densely granulated histological features, carry a low invasive tendency, and achieve higher postoperative biochemical remission rates, representing a molecular subtype with a favorable clinical prognosis (28). Mechanistically, gain-of-function mutations in *USP8* cluster in exon 14 (the 713–720 amino acid region), which disrupts its classical binding motif with the 14-3–3 protein and blocks the normal degradation pathway of EGFR. Sustained EGFR overexpression subsequently drives elevated ACTH secretion. Because *USP8* mutations are frequently accompanied by increased SSTR5 expression, these tumors may exhibit differential sensitivity to pasireotide; however, *USP8* mutation status has not been prospectively validated as a stand-alone biomarker for treatment selection. The USP8–EGFR axis therefore provides a mechanistic rationale for further therapeutic investigation rather than representing an established molecularly guided treatment strategy in PitNETs (29).

## 5 DNA methylation and epigenetic biomarkers

### 5.1 Whole-genome methylation profiling

Genome-wide DNA methylation profiling by Jotanovic et al. demonstrated distinct methylation-based clustering patterns among benign, aggressive, and metastatic PitNETs. Aggressive and metastatic tumors also exhibited more extensive chromosomal copy-number alterations than benign tumors. These findings suggest that DNA methylation profiling may provide candidate molecular information for risk stratification; however, its ability to prospectively predict aggressive clinical behavior or guide individualized adjuvant treatment remains to be established (30).

### 5.2 *CHST7* promoter methylation and lineage-associated tumor biology

*CHST7* promoter methylation represents a lineage-associated epigenetic alteration in PitNETs rather than a circulating biomarker. Dong et al. evaluated *CHST7* expression and promoter methylation in pituitary tumor tissues and demonstrated that its methylation status differed among transcription factor-defined lineages. Notably, SF1-lineage tumors exhibited a higher frequency of *CHST7* promoter methylation than PIT1- and TPIT-lineage tumors. In addition, lower *CHST7* expression was associated with a higher proliferative index, suggesting a potential relationship between *CHST7* dysregulation and tumor proliferative biology. These findings support a role for *CHST7* methylation and expression in lineage differentiation and proliferation, particularly in SF1-lineage PitNETs. However, the available evidence does not establish *CHST7* as a circulating biomarker or as a specific predictor of invasive behavior in non-functioning PitNETs (31).

### 5.3 Integrated epigenomic–transcriptomic signatures associated with PitNET invasiveness

Within the conceptual framework of this review, integrated epigenomic–transcriptomic profiling is relevant primarily to molecular prognostication when the identified alterations are directly associated with invasive behavior. Chen et al. performed reduced representation bisulfite sequencing and RNA sequencing across tumor and blood samples and identified 17 genes showing concurrent alterations in DNA methylation and gene expression in tumor tissue. Among these, *PTPRT* exhibited a particularly strong correlation between intronic DNA methylation and transcript abundance, highlighting the complex relationship between epigenetic regulation and gene expression during PitNET invasion (32).Thus, the principal significance of this dataset in the present review lies in its identification of integrated tissue-based molecular signatures associated with anatomical invasiveness, rather than in the establishment of a new molecular taxonomic class. The circulating DNA methylation component of this study and its potential application as a liquid-biopsy strategy are discussed separately in Section 10.1 to avoid overlap between tissue-based epigenetic biomarkers and circulating biomarkers.

Aydin integrated epigenomic and transcriptomic data across both coding and non-coding regions to dissect disease-specific characteristics, aiming to establish a rational therapeutic framework and drug prioritization strategy for non-functioning PitNETs (NF-PitNETs). By constructing an NF-PitNET protein–protein interaction (PPI) network based on these multi-omics data, the author identified several key hub proteins, including DCC, DLG5, ETS2, FOXO1, HBP1, HMGA2, PCGF3, PSME4, RBPMS, RREB1, SMAD1, SOCS1, SOX2, YAP1, and ZFHX3. Subsequently, profile-based drug repositioning was conducted using these hub genes. Molecular docking analyses identified several compounds with predicted interactions with candidate hub proteins, providing potential candidates for further experimental evaluation in NF-PitNETs. These findings should be regarded as hypothesis-generating and provide a basis for subsequent preclinical validation rather than evidence of established therapeutic efficacy (33).

### 5.4 Epigenomic refinement of PitNET taxonomy

In contrast to the invasion-associated signatures discussed above, the principal relevance of epigenomic profiling in this subsection is taxonomic rather than prognostic. The integration of multi-omics data not only facilitates biomarker discovery but also provides additional insights into PitNET classification. For instance, PPI networks derived from integrated epigenomic, and transcriptomic profiles have identified candidate hub genes in non-functioning PitNETs and provided a basis for exploratory drug-repositioning strategies. In parallel, genome-wide DNA methylation profiling has refined the classification of tumors historically designated as null cell tumors (NCTs). UMAP plotting and methylation-based classification demonstrated that the epigenomes of NCTs with minimal SF1 or TPIT expression were affiliated with the gonadotroph or corticotroph lineages, respectively (34). These findings suggest that DNA methylation profiling may refine lineage assignment in diagnostically ambiguous PitNETs and therefore complement transcription factor-based histopathological classification. Importantly, this application concerns molecular taxonomy and should not be interpreted as evidence that the corresponding methylation classes independently predict invasiveness or clinically defined aggressiveness.

## 6 Transcriptomic and tissue molecular biomarkers associated with invasiveness

*GALR1* (galanin receptor 1) and *PENK* (proenkephalin) have recently emerged as candidate tissue biomarkers associated with invasive behavior in non-functioning PitNETs. Using transcriptomic data from 73 NF-PitNETs, Wu et al. integrated weighted gene co-expression network analysis with multiple machine-learning algorithms, including XGBoost, LASSO, random forest, and support vector machine approaches, and identified *GALR1* and *PENK* as candidate invasion-associated molecules. Both genes were significantly downregulated in invasive tumors, while functional experiments demonstrated that their reduced expression promoted pituitary tumor cell proliferation and invasion. These findings suggest that decreased *GALR1* and *PENK* expression may characterize an invasive phenotype in NF-PitNETs; however, further validation in independent prospective cohorts is required before their clinical prognostic utility can be established (35).

Recent evidence also indicates lineage-dependent transcriptional dysregulation of the mismatch repair (MMR) pathway in invasive PitNETs. Carbonara et al. reported significantly lower *MSH2, MSH6, MLH1*, and *PMS2* transcript levels in invasive than in non-invasive PIT1-lineage tumors, whereas *PMS2* expression was significantly higher in invasive SF1-lineage tumors. These contrasting patterns suggest that the association between MMR gene expression and invasiveness is lineage-dependent rather than uniform across PitNETs. Given the rarity of overt mismatch repair deficiency(MMRd), these transcriptional changes should currently be regarded as exploratory molecular correlates of invasive behavior rather than validated prognostic biomarkers (36).

### 6.1 Tumor microenvironment and immune infiltration characteristics

Single-cell transcriptomic analysis of gonadotroph and lactotroph PitNETs revealed substantial lineage-dependent heterogeneity in the immune microenvironment. In the bulk RNA-seq cohort, proliferating lymphocytes in lactotroph PitNETs were associated with more aggressive tumor behavior. In contrast, single-cell transcriptomic analysis showed that increased abundances of CD8^+^ T cells and natural killer (NK) cells were associated with less invasive phenotypes, with CD8^+^ T-cell enrichment also correlating with reduced invasiveness (37). These findings suggest that lymphoid populations may exert divergent biological effects across PitNET subtypes and provide a basis for further investigation of immune-based therapeutic strategies rather than direct evidence supporting routine immunotherapy (37). Importantly, however, the relationship between the tumor immune microenvironment (TIME) and PitNET aggressiveness remains incompletely defined. Single-cell studies have demonstrated substantial lineage-dependent differences in immune-cell composition and macrophage–tumor interactions, while individual immune-cell populations may show divergent associations with invasive or aggressive tumor phenotypes (37–39). These discrepancies may partly reflect differences in tumor lineage, cohort composition, clinical endpoints, definitions of invasiveness and aggressiveness, and analytical methodology. Accordingly, no reproducible TIME signature has yet been established for predicting invasive behavior or response to immune checkpoint inhibitors (ICIs).Although ICIs have demonstrated clinical activity in selected aggressive or metastatic PitNETs, their optimal timing, treatment sequence, dosing strategy, and predictive biomarkers remain poorly defined (40). Current clinical guidance continues to recommend TMZ monotherapy as first-line chemotherapy for aggressive or metastatic PitNETs (26), whereas ICI therapy should currently be regarded as a later-line option in carefully selected patients (40). Molecular features such as mismatch repair deficiency and increased tumor mutational burden may provide supportive information for patient selection, but their predictive value remains insufficiently validated (26, 40). Therefore, current TIME findings should primarily be regarded as exploratory biological correlates rather than established biomarkers for clinical ICI selection.

## 7 Post-transcriptional modifications

### 7.1 Alternative splicing in PitNETs

Alternative splicing represents a broader layer of post-transcriptional regulation in PitNETs and is not restricted to *NTRK1*/TrkA. Mechanistic evidence from prolactinomas has shown that the recurrent *SF3B1* R625H mutation can induce aberrant RNA splicing. Guo et al. demonstrated that *SF3B1* R625H altered the splicing pattern of multiple transcripts and promoted aberrant splicing and downregulation of *DLG1*, which was accompanied by enhanced cell migration, invasion, and epithelial–mesenchymal transition through PI3K/AKT signaling (41). More recently, Wang et al. identified FUS as another splicing regulator relevant to PIT1-lineage PitNETs. FUS promoted inclusion of exon 3 in *MDM2* pre-mRNA, favoring full-length *MDM2* expression and attenuation of P53-dependent tumor-suppressive signaling; disruption of this FUS-mediated splicing event using splice-switching antisense oligonucleotides suppressed PitNET progression in experimental models (42). Together, these findings indicate that dysregulated splicing in PitNETs may arise from both genetic alterations in core spliceosomal components and abnormal activity of RNA-binding/splicing factors, with downstream effects on tumor proliferation, invasion, and survival.

Alternative splicing of *NTRK1*/TrkA represents another potentially relevant molecular mechanism associated with PitNET invasiveness. Sbaffone et al. (43)evaluated TrkA alternative splicing in 53 PitNETs and identified TrkAIII, an alternative TrkA receptor variant capable of ligand-independent intracellular activation, as the only in-frame potentially oncogenic alternative TrkA isoform detected in these tumors. Among the 53 tumors, 26 were classified as invasive, whereas only two were clinically aggressive and one was metastatic; therefore, the principal findings of this study should be interpreted in relation to anatomical invasiveness rather than clinically defined aggressiveness. TrkAIII mRNA was detected in all invasive PitNETs and in most non-invasive tumors, but the relative TrkAIII/fs-TrkA ratio was significantly higher in invasive than in non-invasive PitNETs. This difference was particularly evident within the PIT1 lineage, whereas no significant invasive versus non-invasive difference was observed among SF1-lineage tumors and the TPIT subgroup was too small for robust statistical comparison. These findings identify enhanced TrkAIII alternative splicing as a candidate molecular feature associated with PitNET invasiveness, particularly in PIT1-lineage tumors, while its widespread expression in non-invasive tumors and the limited number of aggressive or metastatic cases preclude its interpretation as a specific biomarker of clinical aggressiveness. TrkAIII may also represent a potentially targetable molecular alteration, although its diagnostic and therapeutic utility requires validation in larger independent cohorts.

### 7.2 microRNAs

miRNAs have attracted interest as candidate biomarkers in PitNETs because of their relative stability and their potential detectability in tissue or circulating samples. However, to avoid conflating diagnostic utility with prediction of invasive behavior, we restricted this section to miRNAs for which available evidence demonstrates a direct association with clinically relevant adverse phenotypes, including treatment resistance, tumor invasion, or recurrence. The specific dysregulation mechanisms of key miRNAs are outlined below:

miR-34a was retained because its upregulation has been demonstrated in *AIP*-mutated somatotroph PitNETs, a subgroup characterized by treatment-resistant behavior, and experimentally increased miR-34a expression promotes proliferative and migratory phenotypes and contributes to octreotide resistance. Thus, miR-34a may represent a candidate marker of treatment resistance in *AIP*-mutated somatotropinomas rather than a general predictor of PitNET invasiveness (44); miR-137 functions as a tumor-suppressive miRNA and inhibits PitNET cell growth and invasive behavior through suppression of downstream oncogenic signaling, supporting its potential relevance to unfavorable tumor progression (45); miR-423-5p has been proposed as a circulating diagnostic biomarker for somatotroph PitNETs. However, current evidence primarily supports its diagnostic and biological relevance rather than a direct role in predicting clinically aggressive tumor behavior. Its association with clinically defined adverse outcomes, including invasion, recurrence, treatment resistance, or progression, remains insufficiently established (5).

### 7.3 lncRNAs

lncRNAs have increasingly been implicated in PitNET tumorigenesis, cellular proliferation, local invasion, and treatment response, although their direct association with clinically defined tumor aggressiveness remains insufficiently established. Several dysregulated lncRNAs have been reported in PitNETs, including *HOTAIR, H19, RPSAP52, NEAT1*, and *CCAT2.* Rahimian et al. summarized multiple lncRNA alterations in pituitary tumors (6). Whereas subsequent or molecule-specific studies demonstrated dysregulation of *NEAT1* in pituitary adenomas and implicated *CCAT2* in pituitary tumor cell proliferation, migration, and invasion through interaction with *PTTG1* (46, 47). These findings support the biological relevance of lncRNA dysregulation in PitNETs, although their utility as biomarkers of clinically defined aggressiveness remains unestablished. Among these molecules, *H19* has been reported to exert tumor-suppressive effects in lactotroph PitNETs and to enhance dopamine agonist sensitivity through modulation of the mTORC1/4E-BP1 pathway, whereas reduced *H19* expression has been associated with treatment resistance (7). In addition, *LINC00473* was found to be upregulated in invasive compared with non-invasive pituitary tumors. Mechanistically, *LINC00473* functions as a competitive endogenous RNA by suppressing miR-502-3p and consequently increasing *KMT5A* expression, thereby promoting Cyclin D1/CDK2-mediated cell-cycle progression and tumor cell proliferation (48). These findings support a role for specific lncRNAs in PitNET tumor biology, invasiveness, and treatment response; however, none of these lncRNAs has yet been directly validated as a biomarker of clinically defined PitNET aggressiveness. Further studies using well-characterized invasive and non-invasive clinical cohorts with longitudinal follow-up are required to determine their prognostic relevance.

## 8 Protein and immune biomarkers and their microenvironmental effects

### 8.1 PD-L1 expression in PitNETs

PD-L1 expression in PitNETs exhibits substantial heterogeneity across functional and lineage-defined subtypes. Mei et al. demonstrated that functioning pituitary tumors were characterized by greater immune-cell infiltration and increased expression of immunosuppressive checkpoint molecules, including PD-L1, compared with non-functioning tumors, indicating that immune checkpoint profiles vary according to tumor subtype (49). More recent evidence further suggests that PD-L1 expression is preferentially associated with PIT1-lineage PitNETs rather than SF1-lineage tumors (50, 51).

In a cohort of 88 PitNETs classified according to the 2022 WHO framework, Harel et al. demonstrated a significant positive association between PD-L1 expression and PIT1 expression (p < 0.001), particularly in somatotroph, lactotroph, and mammosomatotroph tumors. Conversely, PD-L1 expression was negatively associated with SF1 expression (p < 0.001), with most SF1-lineage tumors showing negative PD-L1 immunostaining (51). These observations are consistent with the immune landscape summarized by Guo et al., which highlights lineage-dependent heterogeneity of the tumor immune microenvironment and suggests relatively greater immune checkpoint activity in PIT1-lineage tumors (50).

Nevertheless, the clinical significance of PD-L1 expression in PitNETs remains incompletely defined. Considerable heterogeneity exists among published studies with respect to cohort composition, antibody clones, immunohistochemical scoring systems, and thresholds for PD-L1 positivity. Moreover, current evidence does not consistently establish PD-L1 as an independent predictor of tumor invasion, recurrence, or clinically aggressive behavior. Therefore, PD-L1 should presently be regarded as a candidate immune biomarker reflecting lineage-specific immune characteristics rather than a validated prognostic biomarker for PitNET aggressiveness.

In a prospective phase II trial of ipilimumab plus nivolumab involving 10 patients with aggressive PitNETs, nine patients were evaluable for the primary endpoint. No objective radiographic responses were observed, although tumor shrinkage occurred in two patients. Exploratory biomarker analyses suggested that TMZ-associated hypermutation and MMRd may represent potential biomarkers associated with ICI response; however, these findings should be interpreted cautiously given the limited sample size (40). Moreover, MMRd appears to be uncommon in PitNETs. In a recent study, complete loss of mismatch repair protein expression, consistent with MMRd, was identified in only 2.2% of the evaluated tumors (36).Available clinical evidence also indicates that MMR status, tumor mutational burden, and PD-L1 immunohistochemical expression do not consistently correlate with clinical responses to ICIs, and responses may occur even in the absence of these candidate biomarkers (52). Therefore, TMZ-associated hypermutation, MMRd, and PD-L1 expression should currently be regarded as exploratory rather than validated predictive biomarkers for ICI response, and their clinical utility for treatment selection requires validation in larger prospective cohorts.

### 8.2 Extracellular matrix and collagen remodeling biomarkers

Collagen possesses remarkable genetic stability and a highly organized spatial architecture, demonstrating potential value across various cancer therapies; it plays multifaceted roles in PitNET progression as both a structural scaffold and a biochemical modulator. In PitNETs, collagen composition and abundance undergo heterogeneous alterations, reflecting the dynamic remodeling of the extracellular matrix by collagen-producing cells. Although collagen remodeling is broadly implicated in tumor progression, its specific contribution to PitNET cell invasion remains uncertain. Nevertheless, several collagen-derived fragments and associated collagenases have been linked to invasive behavior in PitNETs. Additionally, collagen modulates hormone production in PitNETs and can act as a physical and immunological barrier—trapping immune cells and promoting immune evasion through receptor-mediated interactions, which drives the complex immune heterogeneity observed across PitNET subtypes. From a metabolic perspective, collagen can serve as an energy substrate or reprogram metabolic pathways to sustain PitNET proliferation. Future research should focus on clarifying subtype-specific collagen signatures and developing precision therapies to exploit the dual role of collagen as both a pathological driver and a therapeutic target in PitNETs (8). Recent studies further suggest that the extracellular matrix (ECM)-collagen network acts as a crucial driving force for invasive PitNET phenotypes (38).Consistent with this concept, Xie et al. demonstrated that tumor-associated fibroblasts (TAFs) increase ECM stiffness through the secretion of collagen I and collagen III, thereby activating mTOR signaling and promoting epithelial–mesenchymal transition (EMT) (53). Together, these insights establish a well-defined( ECM stiffness–mTOR activation–EMT–invasion) mechanotransduction axis.

## 9 Invasive signaling pathways

### 9.1 The PI3K/AKT pathway

Through differential expression analysis integrating three independent GEO datasets (GSE51618, GSE26966, GSE169498), *SLC7A11* was identified as a candidate invasion-associated molecular marker in integrated transcriptomic analyses of PitNETs. *SLC7A11* is significantly upregulated in invasive tumors; conversely, its knockdown suppresses the proliferation, migration, and invasion of GH3 and GT1–1 cells while inducing apoptosis. Mechanistically, *SLC7A11* drives EMT by activating the PI3K/AKT signaling pathway. *SLC7A11* deficiency leads to a notable reduction in p-PI3K and p-AKT levels, accompanied by the upregulation of E-cadherin and the downregulation of N-cadherin and Vimentin. Furthermore, *SLC7A11* knockdown suppresses PI3K/AKT signaling and modulates EMT-related molecular changes. These findings support *SLC7A11* as a candidate biomarker associated with invasive PitNET biology and identify the *SLC7A11*–PI3K/AKT axis as a potential preclinical therapeutic vulnerability. However, its therapeutic relevance remains investigational, and current evidence does not support *SLC7A11*-directed treatment selection in clinical practice (54).

### 9.2 Bidirectional regulation of the Wnt/β-catenin pathway

The Wnt/β-catenin pathway exerts context-dependent, bidirectional effects on PitNET invasion. Tang et al. (55) showed that *GNAS*-mutant growth hormone–secreting PitNETs exhibit increased maternally expressed gene 3 *(MEG3)* expression and reduced invasive behavior. Functionally, *MEG3* suppresses EMT and tumor cell invasion through inhibition of Wnt/β-catenin signaling, supporting an invasion-suppressive *GNAS*–*MEG3*–Wnt/β-catenin axis in these tumors.

Conversely, Chaudhary et al. (56) demonstrated that DPPA4 enhances Wnt/β-catenin signaling and promotes PitNET cell stemness, proliferation, and invasion. DPPA4 upregulates stemness- and invasion-associated genes and interacts with canonical WNT promoters, thereby enhancing Wnt/β-catenin signaling. Collectively, these findings indicate that Wnt/β-catenin signaling may exert opposing effects on PitNET invasive behavior depending on its upstream molecular regulators: *MEG3* acts as an inhibitory regulator, whereas DPPA4 promotes stemness and invasion. Accordingly, *MEG3* and DPPA4, rather than additional unsupported mediators, are highlighted as the principal context-dependent regulators of this pathway in **Table 2**.These interacting signaling mechanisms are schematically summarized in **Figure 2**.

**Table 2:** Key molecular signaling pathways underlying PitNET invasiveness and emerging targeted therapeutic strategies.

Signaling pathway	Key molecular mediators	Biological role in tumor progression	Potential therapeutic strategy	Ref.
PI3K–AKT signaling	*SLC7A11*, p-PI3K, p-AKT	EMT, cellular proliferation, and invasive behavior	*SLC7A11* inhibition and PI3K/AKT-targeted therapies (preclinical)	(54)
Wnt/β-catenin signaling	*MEG3*, DPPA4, β-catenin	Context-dependent bidirectional regulation of EMT, stemness, and tumor invasion; *MEG3* suppresses whereas DPPA4 enhances Wnt/β-catenin signaling	Preclinical modulation of Wnt/β-catenin signaling; no established clinically actionable strategy	(55, 56)
mTOR signaling	TAFs, ECM stiffness	Mediates mechanotransduction-induced EMT and tumor invasiveness	mTOR inhibitors (preclinical)	(53)
*USP8/EGFR* signaling	*USP8, EGFR*, 14-3–3 proteins	Drives corticotroph PitNET development through enhanced EGFR signaling	*USP8* inhibitors (e.g., DUBs-IN-2) and EGFR inhibitors (e.g., gefitinib) (preclinical)	(28, 29)
INHBA–ACVR1B signaling	CX3CR1^+^ macrophages, INHBA, ACVR1B	Induces apoptosis in *NR5A1*-lineage PitNETs and modulates the tumor microenvironment	ACVR1B agonists (preclinical)	(39)
TRAF6/NF-κB signaling	Exosomal circMFN2/miR-146a-3p axis	Promotes tumor invasion through exosome-mediated activation of NF-κB signaling	circMFN2 silencing and NF-κB inhibition (preclinical)	(58)

EMT, epithelial–mesenchymal transition; ECM, extracellular matrix; EGFR, epidermal growth factor receptor; TAFs, tumor-associated fibroblasts; NF-κB, nuclear factor kappa-B.

**Figure 2:**
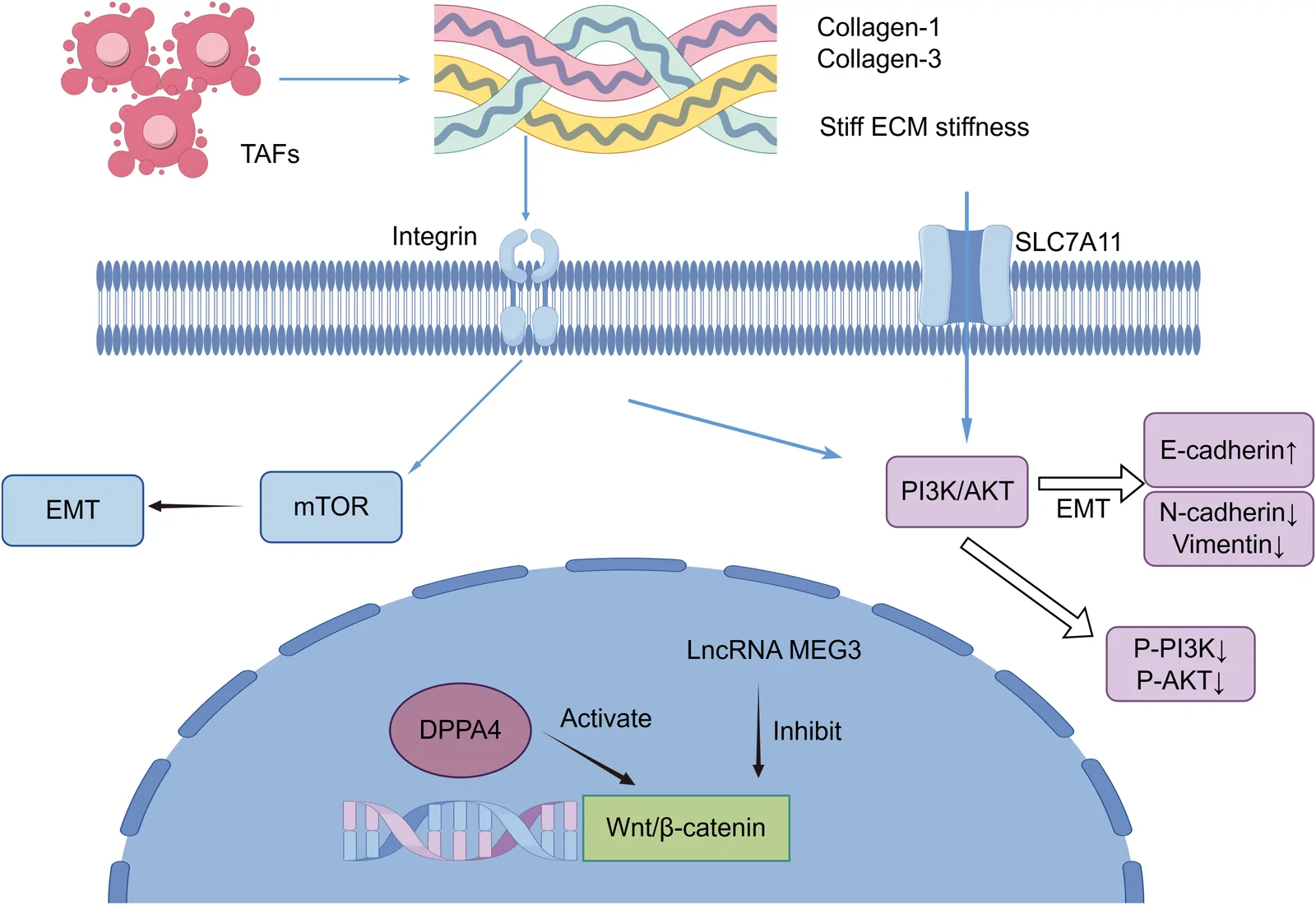
Schematic representation of the molecular mechanisms and signaling pathways governing epithelial-mesenchymal transition (EMT) and invasiveness in PitNETs. Tumor-associated fibroblasts (TAFs) secrete Collagen-1 and Collagen-3, leading to increased extracellular matrix (ECM) stiffness. This stiff ECM activates the transmembrane receptor Integrin, which subsequently triggers the downstream mTOR pathway to promote EMT. Concurrently, stiff ECM crosstalks with the PI3K/AKT axis. On the cell membrane, the novel invasive biomarker *SLC7A11* activates the PI3K/AKT signaling pathway to drive EMT; its deficiency leads to the downregulation of P-PI3K and P-AKT, which is accompanied by the upregulation of E-cadherin and the downregulation of N-cadherin and Vimentin. Within the nucleus, the Wnt/β-catenin pathway undergoes bidirectional regulation: DPPA4 directly binds to the WNT promoter to activate this pathway, promoting tumor cell stemness and invasion, whereas the long non-coding RNA *MEG3* (LncRNA *MEG3*) acts as an endogenous inhibitor to suppress Wnt/β-catenin signaling, thereby restraining the invasive phenotype of PitNETs. This schematic was designed by the authors based on the mechanistic evidence summarized in the cited studies, with graphical resources and design tools provided by Figdraw.

### 9.3 Signaling axes mediated by circulating metabolites

Beyond traditional intracellular signal transduction, circulating metabolites also participate in regulating tumor invasiveness. Multi-omics integrated analyses revealed that phosphatidylinositol (PI) is the most significantly upregulated metabolite in the serum of patients with invasive PitNETs. Elevated PI directly promotes tumor cell proliferation and metastasis via the *PITPNM1/AKT/ERK/POU1F1* signaling axis, establishing a novel bridge between systemic humoral metabolism and localized tumor invasion. Xu et al. using combined proteomics and serum metabolomics, confirmed that serum PI represents the most prominent upregulated metabolic product in invasive cohorts, providing a valuable proof-of-concept for predicting tumor invasiveness through serum PI testing (57). Recent advances in transcriptomic, epigenetic, and tumor-microenvironment studies have substantially expanded our understanding of the molecular mechanisms underlying PitNET invasiveness. Multiple signaling pathways have been implicated in epithelial–mesenchymal transition, tumor stemness, mechanotransduction, and immune modulation. Importantly, mechanistic targetability should not be equated with clinical actionability: most of the pathways discussed below currently represent experimental or preclinical therapeutic hypotheses rather than established treatment targets. Therapeutic decisions for aggressive PitNETs should continue to be based primarily on documented disease progression, previous treatment response, and established multidisciplinary management, with TMZ remaining the recommended first-line chemotherapy for aggressive or metastatic PitNETs according to current clinical guidance (20). The key signaling networks and their investigational therapeutic implications are summarized in **Table 2**.

## 10 Emerging translational approaches for PitNET risk assessment

### 10.1 Liquid biopsy and circulating biomarkers

Liquid biopsy provides a minimally invasive and potentially repeatable approach for characterizing tumor-derived molecular alterations and may complement conventional tissue-based assessment during the diagnosis and longitudinal surveillance of PitNETs. To reduce thematic overlap between biomarker-specific sections, circulating DNA methylation, circulating non-coding RNAs, extracellular vesicles, and circulating tumor cells (CTCs) are considered together in this section rather than being discussed separately within the corresponding tissue-based molecular categories (5).Among the most promising preliminary approaches is plasma cell-free DNA (cfDNA) methylation profiling. Chen et al. integrated reduced representation bisulfite sequencing and transcriptomic analyses of invasive and non-invasive pituitary tumors and subsequently evaluated tumor-associated DNA methylation alterations in plasma samples. Three methylation markers, *MIR4535*, *SLC8A1-AS1*, and *TTC34*, collectively discriminated invasive from non-invasive PitNETs with an area under the receiver operating characteristic curve (AUC) of 0.980 and differentiated non-invasive tumors from healthy controls with an AUC of 0.990 (32). These findings provide proof-of-concept that tumor-associated epigenetic alterations can be detected in peripheral blood and may potentially support non-invasive assessment of PitNET biological behavior. These findings support the potential of this cfDNA methylation panel as a candidate biomarker; however, its clinical utility and prognostic performance require confirmation in independent prospective cohorts.

Extracellular vesicles, particularly exosomes, represent another potential source of circulating tumor-derived molecular information. Exosomal cargo includes non-coding RNAs, messenger RNAs, proteins, and other biologically active molecules that may reflect tumor phenotype while also participating in intercellular communication (5, 7). For example, exosomal circMFN2 has been reported to promote pituitary tumor cell proliferation and migration through regulation of the miR-146a-3p/TRAF6/NF-κB signaling axis (58). Other candidate exosomal molecules, including miR-486-5p, miR-423-5p, *CDK6*, *RHOU*, and *MMP1*, have been associated with recurrence, hormone secretion, cellular proliferation, invasion, or angiogenesis in individual studies and reviews (5, 7). However, the clinical endpoints evaluated across these studies are heterogeneous, and associations with tumor invasion, recurrence, treatment resistance, and clinically defined aggressiveness should not be considered interchangeable. Accordingly, circulating exosomal molecules remain exploratory biomarkers rather than established predictors of invasive PitNET behavior.

Other liquid-biopsy components remain less extensively characterized in PitNETs. CTCs are particularly difficult to detect because of their extremely low abundance, whereas tumor-derived cfDNA may constitute only a small fraction of total circulating DNA. In addition, variability in pre-analytical processing, extracellular-vesicle isolation, RNA normalization, and analytical platforms limits direct comparison between studies (5, 7). Therefore, although liquid biopsy provides an attractive strategy for repeated and minimally invasive tumor monitoring, current evidence does not support its replacement of conventional radiological, histopathological, or longitudinal clinical assessment. Prospective multicenter studies with standardized sampling and analytical protocols will be required to determine whether circulating biomarkers can provide incremental prognostic information beyond established clinicopathological parameters. These emerging biomarkers, together with their reported clinical applications and current limitations, are summarized in **Table 3**.

**Table 3:** Emerging circulating biomarkers and their potential clinical applications in liquid biopsy for pituitary neuroendocrine tumors (PitNETs).

Biomarker category	Representative biomarkers	Potential clinical applications	Current limitations
ctDNA	Methylated *MIR4535* and *SLC8A1-AS1*	Identification of invasive PitNETs (AUC = 0.980); potential tool for risk stratification	Extremely low abundance in circulation, requiring highly sensitive detection platforms
ncRNA	miR-34a, miR-137, miR-423-5p	Prediction of therapeutic response; molecular subtype discrimination; disease monitoring	Lack of standardized protocols for sample processing, detection, and data normalization
CTCs	Not yet well characterized in PitNETs	Real-time assessment of tumor dynamics and treatment response	Rarely detected in PitNET patients, limiting clinical applicability
Exosomes	Tumor-derived nucleic acids, proteins, and metabolites	Multi-omics profiling; biomarker discovery; non-invasive disease monitoring	Isolation and purification methods remain technically challenging and insufficiently standardized

PitNET, pituitary neuroendocrine tumor; ctDNA, circulating tumor DNA; ncRNA, non-coding RNA; CTCs, circulating tumor cells; AUC, area under the receiver operating characteristic curve.

### 10.2 MRI radiomics and deep-learning-based imaging biomarkers

Radiomics has emerged as a complementary approach for extracting high-dimensional quantitative information from routinely acquired magnetic resonance imaging (MRI). In contrast to conventional radiological evaluation, which primarily characterizes macroscopic anatomical features such as tumor size and cavernous sinus invasion, radiomic analysis can quantify tumor shape, signal intensity, texture, and spatial heterogeneity that may not be readily appreciable by visual assessment. These imaging-derived features therefore provide a potential non-invasive bridge between anatomical phenotype and underlying tumor biology.

Recent studies have demonstrated encouraging performance of radiomics for the preoperative assessment of PitNET invasiveness. Calandrelli et al. retrospectively evaluated 200 patients with macro-PitNETs using radiomic features extracted from contrast-enhanced T1-weighted and T2-weighted MRI. An XGBoost model combining five radiomic features from both sequences achieved an AUC of 0.85 (95% CI, 0.75–0.95) for distinguishing invasive from non-invasive tumors according to the Trouillas classification (59). These findings indicate that quantitative measures of tumor shape, texture, and intratumoral signal heterogeneity may complement conventional MRI-based assessment of invasive growth. However, the single-center retrospective design, manual segmentation, and absence of external validation currently limit the generalizability of this approach.

More recently, Wang et al. developed a deep learning radiomics framework using data from 1,089 patients across three medical centers (60). The model integrated automated tumor segmentation and deep-learning-based feature extraction to generate a radiomics score that was strongly associated with conventional Knosp and Hardy–Wilson invasion classifications. Importantly, the deep-learning radiomics score also outperformed these conventional anatomical grading systems in predicting tumor recurrence and showed associations with Ki-67, P53 expression, macrophage-related pathological features, and molecular pathways including MAPK and TGF-β signaling (60). This study suggests that radiomics may extend beyond anatomical invasion assessment and potentially capture imaging phenotypes associated with adverse biological behavior. Integration of radiomics with molecular, pathological, and circulating biomarkers may ultimately provide a more comprehensive framework for individualized risk stratification.

### 10.3 Tumor stemness and pituitary tumor stem-like cells

Tumor stemness represents another emerging dimension of PitNET biology that may contribute to tumor initiation, cellular plasticity, local invasion, therapeutic resistance, and recurrence. Putative pituitary tumor stem-like cells have been identified in human and experimental pituitary tumors using combinations of stemness-associated markers, including SOX2, CD133, Nestin, OCT4, and NANOG. A recent systematic review of 34 studies further highlighted the involvement of developmental and oncogenic signaling networks, including Wnt/β-catenin, Notch, Sonic Hedgehog, PI3K/AKT/mTOR, and related pathways, in the maintenance of stem-like phenotypes in pituitary tumors (61). Nevertheless, several primary studies provide more direct evidence linking stem-like populations to invasive phenotypes.

In human pituitary tumor specimens, Chen et al. identified neurosphere-forming stem/progenitor-like cells and reported significantly higher CD133 expression in invasive than in non-invasive pituitary tumors, providing early clinical evidence of an association between stem-like features and anatomical invasiveness (62). Peverelli et al. subsequently examined 46 non-functioning pituitary tumors and found that approximately 70% generated progenitor/stem-like spheres; importantly, sphere-forming capacity was associated with invasive tumor behavior (OR = 3.96, 95% CI 1.05–14.88), linking a functional stem-like phenotype, rather than marker expression alone, to invasiveness (63).

Functional studies provide complementary evidence. Mertens et al. identified a side population enriched for stemness-associated and epithelial–mesenchymal transition (EMT)-related features in human pituitary tumors. In experimental models, this population exhibited greater tumorigenic potential, while inhibition of CXCR4 signaling reduced EMT-associated cell motility and xenograft tumor growth, suggesting a functional connection between stem-like cellular states and invasive programs (64). Similarly, Würth et al. isolated stem-like cultures from human pituitary tumors that expressed OCT4, SOX2, CD133, and Nestin and exhibited sustained self-renewal and differentiation capacity. Although these cells did not demonstrate conventional tumorigenicity in mice, pituisphere-derived cells engrafted in zebrafish and induced pro-angiogenic and invasive responses, further supporting a potential contribution of stem-like populations to adverse tumor behavior (65).

Recent mechanistic evidence provides a more direct link between stemness-associated programs and adverse PitNET phenotypes. Chaudhary et al. demonstrated that developmental pluripotency-associated 4 (DPPA4), a stem-cell regulatory protein, enhances PitNET cell stemness, proliferation, migration, and tumorigenic potential (56). DPPA4 increased the expression of stemness-associated factors and promoted canonical Wnt/β-catenin signaling, whereas genetic or pharmacological interference with this pathway attenuated several invasive cellular phenotypes. These findings support a potential DPPA4–Wnt/β-catenin–stemness axis linking developmental transcriptional programs to invasive tumor biology (56). Importantly, however, these mechanistic observations should not be interpreted as establishing DPPA4 or other stemness-associated molecules as clinically validated prognostic biomarkers. Independent evidence has emerged for a CDK8–SOX2 regulatory axis. Xie et al. reported that CDK8 expression in clinical pituitary tumor specimens correlated with Knosp grade and showed that CDK8 phosphorylates SOX2, thereby reducing its HERC5-mediated degradation and maintaining stem-like self-renewal. CDK8 inhibition impaired tumorsphere formation in patient-derived pituitary tumor stem-like cells and suppressed tumor-cell proliferation and xenograft growth, providing an additional mechanistic connection between stemness maintenance and an invasion-associated clinical phenotype (66).

Most recently, Yuan et al. performed RNA sequencing of human growth hormone-secreting pituitary tumor stem-like cells and matched differentiated tumor cells and identified CXCR4 as a central stemness-associated regulator. Functional suppression of CXCR4 impaired self-renewal and differentiation of human pituitary tumor stem-like cells and reduced proliferation, migration, invasion, angiogenesis, and EMT-related phenotypes in experimental models (67). These findings extend earlier CXCR4 observations by directly integrating stem-like cell transcriptomics with functional invasion assays.

However, the relationship between stemness-associated markers and anatomical invasiveness is not uniform across studies. Basaran et al. evaluated CD133, OCT4, SOX2, and Nestin by immunohistochemistry in 48 pituitary tumors classified according to radiological invasiveness and found no significant association between the expression of these individual markers and invasive versus non-invasive status (68). This apparent discrepancy with functional stem-like cell studies underscores an important distinction: expression of an isolated stemness-associated marker is not equivalent to demonstration of a functional tumor stem-like state. Differences in tumor lineage, marker definitions, invasion criteria, sampling, and experimental methodology may further contribute to the heterogeneous findings.

The clinical interpretation of pituitary tumor stem-like cells also remains challenging because the field is methodologically heterogeneous. Stem-like populations have been defined using different combinations of surface markers, sphere-formation assays, side-population approaches, primary tumor cultures, and experimental animal models, while several putative stem-cell markers, particularly SOX2, are also expressed by normal pituitary progenitor populations (61). Consequently, no individual stem-cell marker or composite stemness signature has yet been prospectively validated for predicting clinically defined PitNET aggressiveness. Future studies integrating single-cell sequencing, spatial transcriptomics, lineage-tracing approaches, and longitudinal clinical follow-up will be required to determine whether stem-like populations represent true drivers of invasive progression, biomarkers of tumor plasticity, or therapeutically targetable subpopulations.

Taken together, the available primary evidence supports a biologically plausible association between functional stem-like states and invasive or otherwise adverse PitNET phenotypes, particularly through CXCR4/EMT, DPPA4–Wnt/β-catenin, and CDK8–SOX2 signaling. However, the evidence remains heterogeneous and should not be interpreted as establishing a clinically validated stemness biomarker for invasiveness. In particular, functional properties such as sphere formation, self-renewal, EMT activation, or experimentally demonstrated invasive capacity appear more informative than the immunohistochemical expression of individual markers such as SOX2, CD133, Nestin, or OCT4 alone. No individual marker or composite stemness signature has yet been prospectively validated for predicting anatomical invasiveness, recurrence, or clinically defined aggressiveness. Future studies should integrate functional stem-cell assays, single-cell and spatial transcriptomics, lineage-resolved molecular profiling, and longitudinal clinical outcomes to determine whether specific stem-like populations provide incremental prognostic information beyond established clinicoradiological and pathological parameters.

## 11 Conclusion

Current evidence indicates that molecular prognostication in PitNETs is moving beyond conventional histopathological assessment toward biologically informed risk stratification. Among the molecular alterations reviewed, *TP53, ATRX*, and *SF3B1* currently represent some of the better-characterized candidate tissue markers associated with invasive or unfavorable tumor behavior. *TP53* alterations and ATRX loss are particularly enriched in invasive or treatment-refractory corticotroph PitNETs and may reflect genomic instability and clonal evolution, whereas *SF3B1* mutations in lactotroph tumors have been associated with metastatic disease and adverse survival outcomes. These alterations are therefore promising tissue-based risk-enrichment markers, although none has yet demonstrated sufficient prospective performance to serve as an independent predictor of clinically defined aggressiveness. Additional phenotype-specific candidates may provide complementary information regarding PitNET biological behavior. In particular, increased alternative TrkAIII splicing has been associated with anatomical invasiveness, especially in PIT1-lineage tumors, although its presence in most non-invasive tumors limits its current specificity as a stand-alone marker. Treatment-response-associated non-coding RNAs, including *miR-34a* and *H19*, represent a separate class of exploratory biomarkers.

Liquid-biopsy and microenvironmental biomarkers further broaden the potential scope of non-invasive risk assessment. In particular, cfDNA methylation signatures involving *MIR4535, SLC8A1-AS1*, and *TTC34* showed high discriminatory performance for invasive PitNETs in the initial study cohort and warrant further evaluation as candidate markers in longitudinal studies. However, their ability to predict clinically defined aggressive behavior, rather than anatomical invasion alone, remains to be established. Similarly, circulating non-coding RNAs, extracellular-vesicle cargo, metabolic signatures, and tumor immune-cell profiles—including proliferating lymphocytes and CD8T^+^-cell/NK-cell infiltration—provide biologically plausible indicators of tumor behavior but remain exploratory. Thus, biomarkers associated predominantly with invasion, molecular subtype, or treatment response should not be interpreted as validated predictors of aggressiveness without longitudinal evidence demonstrating rapid progression or clinically relevant progression despite optimal standard therapy.

Overall, no single molecular biomarker currently has sufficient validation to independently predict invasive PitNET behavior. The most promising near-term strategy is therefore to integrate the above candidate markers with established clinical, radiological, and pathological variables, including longitudinal tumor growth, treatment response, Knosp/Hardy features, lineage classification, and proliferative indices. Future studies should prioritize prospective multicenter validation, standardized analytical workflows, and clinically defined endpoints that distinguish invasion, recurrence, treatment resistance, and true aggressiveness. Such prospectively validated clinicoradiological–pathological–molecular models may ultimately improve individualized risk stratification and help inform surveillance and management strategies for patients at risk of invasive PitNET progression.
